
JOURNAL INFORMATION
==============================
NLM Title Abbreviation: ScientificWorldJournal
Journal ID: ScientificWorldJournal
NLM Journal ID: 101131163
Journal ID: TSWJ

The Scientific World Journal

ISSN: 2356-6140
EISSN: 1537-744X
Publisher: Wiley

ARTICLE INFORMATION
==============================
PMCID: PMC6009758
PMID: 29973789
DOI: 10.1100/tsw.2002.15
Article ID: 745898
Article version: 1
Subjects: Short Report

Progress in AB Initio Protein Structure Prediction

Baker David *
Department of Biochemistry, University of Washington, 1959 N.E. Pacific, Box 357350, Seattle, WA 98195-7350, USA
*David Baker: dabaker@u.washington.edu
Publication date: 2002
Electronic publication date: 2002 May 11
Volume: 2
First page: 31
Last page: 31
Copyright: Copyright © 2002 David Baker.
Copyright year: 2002
License: This is an open access article distributed under the Creative Commons Attribution License, which permits unrestricted use, distribution, and reproduction in any medium, provided the original work is properly cited.
License URL: https://creativecommons.org/licenses/by/3.0/

==============================
Advances in Gene Technology: The Genome and Beyond –
Structural Biology for Medicine (Proceedings of the 2002 Miami
*Nature Biotechnology* Winter Symposium)
*TheScientificWorld* 2002, 2(S2), 31
ISSN 1532-2246; DOI 10.1100/tsw.2002.15

### PROGRESS IN *AB INITIO* PROTEIN STRUCTURE PREDICTION

David Baker
Department of Biochemistry, University of Washington, 1959 N.E. Pacific, Box 357350, Seattle, WA 98195-7350
dabaker@u.washington.edu

I will discuss the application of the Rosetta method for *ab nitio* protein structure prediction to the prediction of protein structure and function on the genome scale. Rosetta is based on a picture of protein folding in which short segments of the chain flicker between different local structures consistent with the sequence and folding occurs when the segments adopt structures and orientations such that low free energy interactions are made throughout the chain. Results from the CASP4 protein structure prediction experiment and larger scale in house tests suggest that for roughly 50% of domains less than 150 residues in length at least one of the top five most commonly generated Rosetta models has sufficient similarity to the native structure to identify it in a structure based search of the PDB. While not suitable for applications such as ligand docking which require atomic level resolution, functional insights can be obtained from Rosetta models through structure based searches of the PDB and characterization of the positions of evolutionarily conserved residues. Rosetta models have been produced for all PFAM families less than 150 residues in length which do not currently have a representative of known structure and are now being generated for proteins in the yeast genome.

### REFERENCES

1. Baker, D. and Sali, A. (2001) Science 294(5540), 93–96.
2. Simons, K.T., Bonneau, R., and Baker, D. (2001) J. Mol. Biol. 306, 1191–1199.
3. Simons, K.T., Bonneau, R., Ruczinski, I., and Baker, D. (1999) Proteins 37(S3), 171–176.
